# Mutational Landscape of PI3K-AKT-mTOR Pathway in Breast Cancer: Implications for Targeted Therapeutics

**DOI:** 10.7150/jca.52993

**Published:** 2021-05-27

**Authors:** Weikai Xiao, Guochun Zhang, Bo Chen, Xiaoqing Chen, Lingzhu Wen, Jianguo Lai, Xuerui Li, Min Li, Hao Liu, Jing Liu, Han Han-Zhang, Analyn Lizaso, Ning Liao

**Affiliations:** 1 Department of Breast Cancer, Cancer Center, Guangdong Provincial People's Hospital, Guangdong Academy of Medical Sciences, Guangzhou, China.; 2 Department of Breast, Foshan Women and Children Hospital, Foshan, China.; 3 Burning Rock Biotech, Guangzhou, China.

**Keywords:** Breast cancer, PI3K-AKT-mTOR pathway, gene alteration, molecular subtypes, prognosis

## Abstract

**Background:** Comprehensive analysis of PI3K-AKT-mTOR pathway gene alterations in breast cancer may be helpful for targeted therapy.

**Methods:** We performed targeted sequencing using a panel of 520 cancer-related genes to investigate gene alterations in the PI3K-AKT-mTOR pathway from 589 consecutive Chinese women diagnosed with stage I-III breast cancer. Analyses of overall survival (OS) were performed using the publicly available clinical and genomic data from METABRIC.

**Results:** PI3K-AKT-mTOR pathway gene alterations were detected in 62.6% (369/589) of our cohort. The most commonly altered genes were *PIK3CA* (45%), *PTEN* (7.5%), *AKT1* (5.9 %), *PIK3R1* (2.7%), and *PIK3CG* (2%). Four *PIK3CA* mutations (E545K, H1047R, E542K, and H1047L) were detected in all the breast cancer molecular subtypes. Seven *PIK3CA* mutations (E545G, E418_L422delinsV, E726K, E110del, G1049R, G118D, and D350G) were only detected in HR^+^ subtypes. Two *PIK3CA* mutations (C420R and N345K) were only detected in non-triple-negative subtypes. Most cases with *PTEN* mutation were HR^+^/HER2^-^ subtype (77.3%), followed by triple-negative subtype (18.2%). In the METABRIC breast cancer dataset, no significant OS difference was observed between the *PIK3CA*-mutant and wild-type groups. However, patients with multiple *PIK3CA* mutations (mOS: 131 vs. 159 months, *P*= 0.029), or *PIK3CA* mutations located in the* C2* domain had significantly shorter OS (mOS, 130 vs. 154 months, *P*=0.020) than those without the mutations.

**Conclusions:** Our study reveals the heterogeneity in PI3K-AKT-mTOR pathway among the breast cancer molecular subtypes in our cohort. Moreover, the number and specific sites of *PIK3CA* mutations have distinct prognostic impact.

## Introduction

The PI3K-AKT-mTOR pathway is among the most common activation signals in various cancer types 1-3. This pathway is involved in the regulation of cell proliferation, apoptosis, metabolism, migration, and invasion 4, 5. The activation of this pathway begins with the binding of the ligand to the corresponding receptor tyrosine kinase, phosphoinositide 3-kinase (PI3K). The regulatory subunit of PI3K becomes phosphorylated and leads to the activation of p110, its catalytic subunit. The activated PI3K produces phosphatidylinositol 3, 4, 5-triphosphate (PIP_3_), a lipid second messenger, and leads to further activation of downstream effectors such as protein kinase B (AKT) and mammalian target of rapamycin (mTOR) 4. On the other hand, phosphatase and tensin homolog (PTEN) counteracts the function of PI3K by dephosphorylating PIP_3_ and is regarded as the major natural inhibitor of the PI3K-AKT-mTOR pathway 4.

Breast cancer is the most common malignancy in women worldwide. The PI3K-AKT-mTOR signaling pathway plays an important role in the development of breast cancer 6-9. Various inhibitors have been developed for targeting the three major components of the PI3K-AKT-mTOR pathway and have shown promising therapeutic effects in breast cancer 7, 10. For example, a specific inhibitor of PI3Kα, alpelisib (BLY719) in combination with fulvestrant in the SOLAR-1 clinical trial had shown encouraging results by prolonging progression-free survival (PFS) in patients with advanced *PIK3CA*-mutant breast cancer as compared with fulvestrant alone 11. Moreover, the results of the PAKT trial confirmed that the addition of the AKT inhibitor capivasertib to first-line paclitaxel therapy can significantly prolong PFS and overall survival (OS) in patients with triple-negative breast cancer 12. Similarly, combination therapy with mTOR inhibitor and exemestane was also shown to significantly prolong the OS of patients with hormone receptor (HR)-positive metastatic breast cancer 13, 14. Therefore, targeting the PI3K-AKT-mTOR signaling pathway has become promising therapeutic option in the clinical treatment of breast cancer. With the advent of novel promising PI3K-AKT-mTOR pathway inhibitors, it is important to identify the patients who can benefit from these inhibitors. The comprehensive analysis of the PI3K-AKT-mTOR pathway gene aberrations in breast cancer may help choose the proper inhibitors to precisely treat certain molecular subset of patients. Next-generation sequencing (NGS) technology has made it possible to quickly and accurately identify such genetic aberrations. In this study, we systematically analyzed the genetic aberrations in the PI3K-AKT-mTOR pathway in a large cohort of 589 Chinese women with early-stage breast cancer by NGS methods.

## Materials and methods

### Patients and specimens

Women diagnosed with breast cancer (American Joint Committee on Cancer (AJCC) Tumor, Node, Metastasis (TNM) stage IA to III) at the Department of Breast Cancer in Guangdong Provincial People's Hospital who had tissue samples submitted for NGS were included in this study. Breast tumor tissues were obtained by surgical resection and processed as formalin-fixed paraffin-embedded (FFPE) blocks. Expression status for estrogen receptor (ER), progesterone receptor (PR), and human epidermal growth factor receptor 2 (HER2) were examined according to the guidelines of the American Society of Clinical Oncology (ASCO) /College of American Pathologists (CAP) 15. All tumor samples were reviewed by breast pathologists, and clinical data were obtained from medical records. This study was approved by the institutional review board of the Guangdong Provincial People's Hospital. Written informed consent were provided by all patients.

### Targeted sequencing and analysis

Sample processing, NGS library construction and subsequent sequencing analysis were performed at Burning Rock Biotech, a CAP-accredited and Clinical Laboratory Improvement Amendments (CLIA)-certified clinical laboratory as described previously 16. In brief, tumor tissue DNA were extracted from FFPE tumor samples using QIAamp DNA FFPE tissue kit (Qiagen, Hilden, Germany), according to the manufacturer's instructions. The DNA concentration was quantified by Qubit 2.0 fluorimeter with the dsDNA high sensitivity assay kit (Life Technologies, Carlsbad, CA, USA). A minimum of 50 ng DNA was used for the NGS library construction. Indexed samples were sequenced on an Illumina NextSeq 500 instrument (Illumina, Inc., Hayward, CA, USA) with paired-end reads and average sequencing depth of 1,000X using a commercially available panel targeting 520 cancer-related genes, spanning 1.64 megabases (Mb) of the human genome (OncoScreen Plus, Burning Rock Biotech, Guangzhou, China). The panel was designed to capture whole exons of 312 genes and critical exons, introns, and promoter regions of the remaining 208 genes. The panel interrogates genes included in the PI3K-AKT-mTOR pathway, including all exons of *AKT1-3*, *MTOR*, *PIK3CA/B/G*, *PIK3R1-2*, and *PTEN* and critical exons, introns, and promoter region of *PIK3C2B/G*, *PIK3C3*, *PIK3CD*, and *PIK3R3*, which allows accurate detection of single nucleotide variants (SNV) and copy number variants. Paired white blood cells from each patient were used to filter out germline mutations. Sequencing data were analyzed using proprietary computational algorithms optimized for somatic variant calling.

### Sequence data analysis

Sequence data were mapped to the human genome (hg19) using Burrows-Wheeler Aligner v.0.7.10. Local alignment optimization, variant calling, and annotation were performed using GATK v.3.2 and VarScan v.2.4.3. Variants were filtered using the VarScan fpfilter pipeline, with loci with depth less than 100 filtered out. At least 5 supporting reads were required for insertion-deletion variants; while 8 supporting reads were required for SNVs to be called. According to the ExAC, 1000 Genomes, dbSNP, and ESP6500SI-V2 databases, variants with population frequency over 0.1% were grouped as single nucleotide polymorphisms and excluded from further analysis. The remaining variants were annotated with ANNOVAR and SnpEff v.3.6. DNA translocation analysis was performed using Factera v.1.4.3. Copy number variation was detected using in-house bioinformatics pipeline based on the depth of coverage data of capture intervals.

### Statistical analysis

Descriptive statistics were used to summarize patient features and sequencing results, including median and range for categorical variables, mean, and standard deviation for continuous variables. Chi-square test or Fisher's exact test was used to compare the differences in demographic, clinical, and pathological characteristics in different groups. All statistical tests were two-way. P value of less than 0.05 was considered significant.

Kaplan-Meier analysis with log-rank test was performed to analyze OS data obtained from the Molecular Taxonomy of Breast Cancer International Consortium (METABRIC) 17. OS was defined from the date of initial diagnosis until death. A total of 2,388 breast cancer patients with TNM stage I-III from the METABRIC dataset were included in the survival analysis, including TNM stage I (n = 214), stage II (n = 976), and stage III (n = 1198). P-values were adjusted with clinical data including age, tumor stage, histological subtype, menopausal status, therapy received (radiotherapy, hormone therapy, or chemotherapy).

## Results

### Baseline patient characteristics

Our study included 589 Chinese women between the ages of 22 and 85 years who were diagnosed with stage I-III breast cancer. The clinicopathological characteristics of the patients are listed in Table S1. A total of 321 (54.5%) patients were HR^+^/HER2^-^, 111 (18.8%) were HR^+^/HER2^+^, and 64 (10.9%) were HR^-^/HER2^+^, while 68 (11.5%) had triple-negative breast cancer (TNBC, HR^-^/HER2^-^).

### Somatic mutations of PI3K-AKT-mTOR pathway

**Figure 1:**
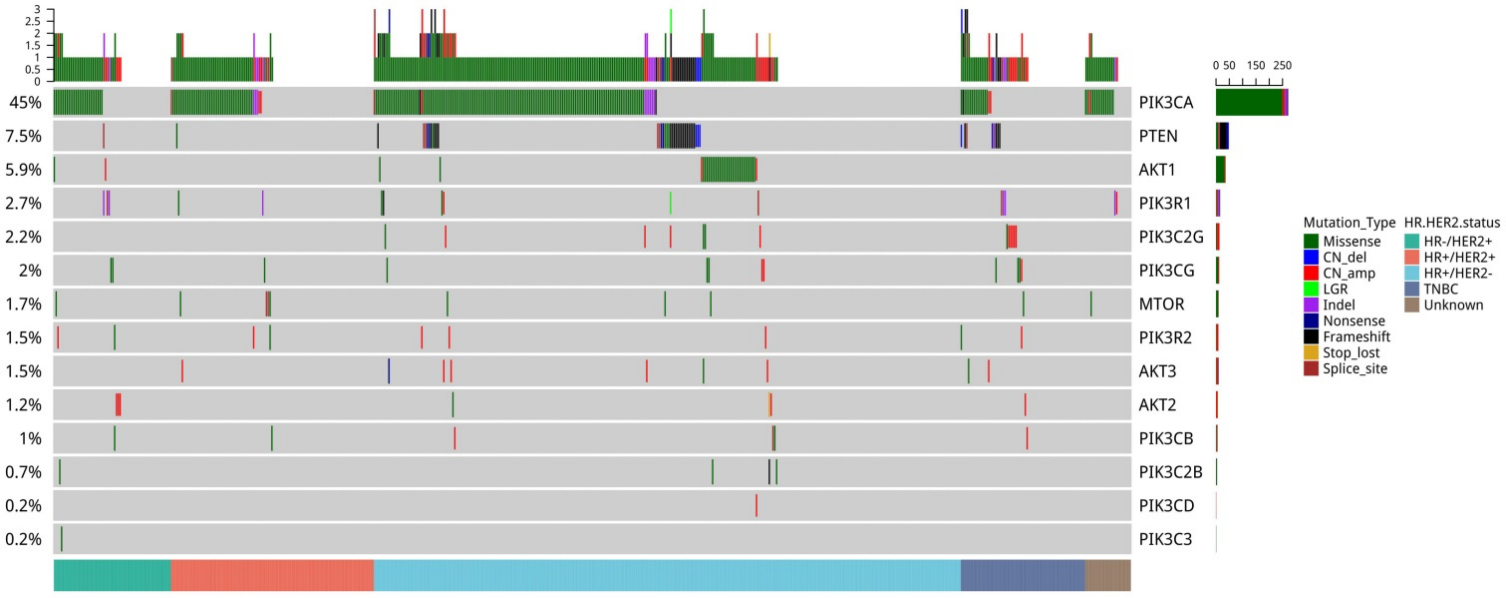
Mutation profile of genes in the PI3K-AKT-mTOR pathway of 589 Chinese patients with breast cancer. Tumor samples were grouped according to molecular subtype: HR^+^/HER2^-^ (n = 321), HR^+^/HER2^+^ (n = 111), HER2-rich (n = 64), and TNBC (n = 68) as indicated by the annotation at the bottom. The mutation frequency for each gene is shown on the left. Colors indicate the mutation types.

To understand the mutational pattern of genes involved in the PI3K-AKT-mTOR pathway, we analyzed the somatic mutation landscape of our breast cancer cohort. In total, 62.6% (369/589) of patients had at least one mutation in the PI3K-AKT-mTOR pathway. Among all the genes analyzed, *PIK3CA* was the most frequently mutated gene (45.0%, 265/589), followed by *PTEN* (7.5%, 44/589), *AKT1* (5.9%, 35/589), *PIK3R*1 (2.7%, 16/589), and *PIK3CG* (2.0%, 12/589) (Figure 1). Mutations in other genes were less common. In the METABRIC cohort, *PIK3CA* was also the most frequently mutated gene (41.3%, 1035/2,509), and multiple *PIK3CA* mutations accounted for 15.5% (160/1,035). The frequencies of copy number amplifications in *PIK3C2B* and *AKT3* in the METABRIC cohort were 21.6% and 20%, respectively, which were significantly higher than in our cohort. Subsequently, the mutation rates were 5.9% for *PTEN*, 5.0% for *AKT1*, and 2.7% for *PIK3R1* in the METABRIC cohort, which were similar to our cohort (Figure S1).

### PIK3CA mutations

**Figure 2:**
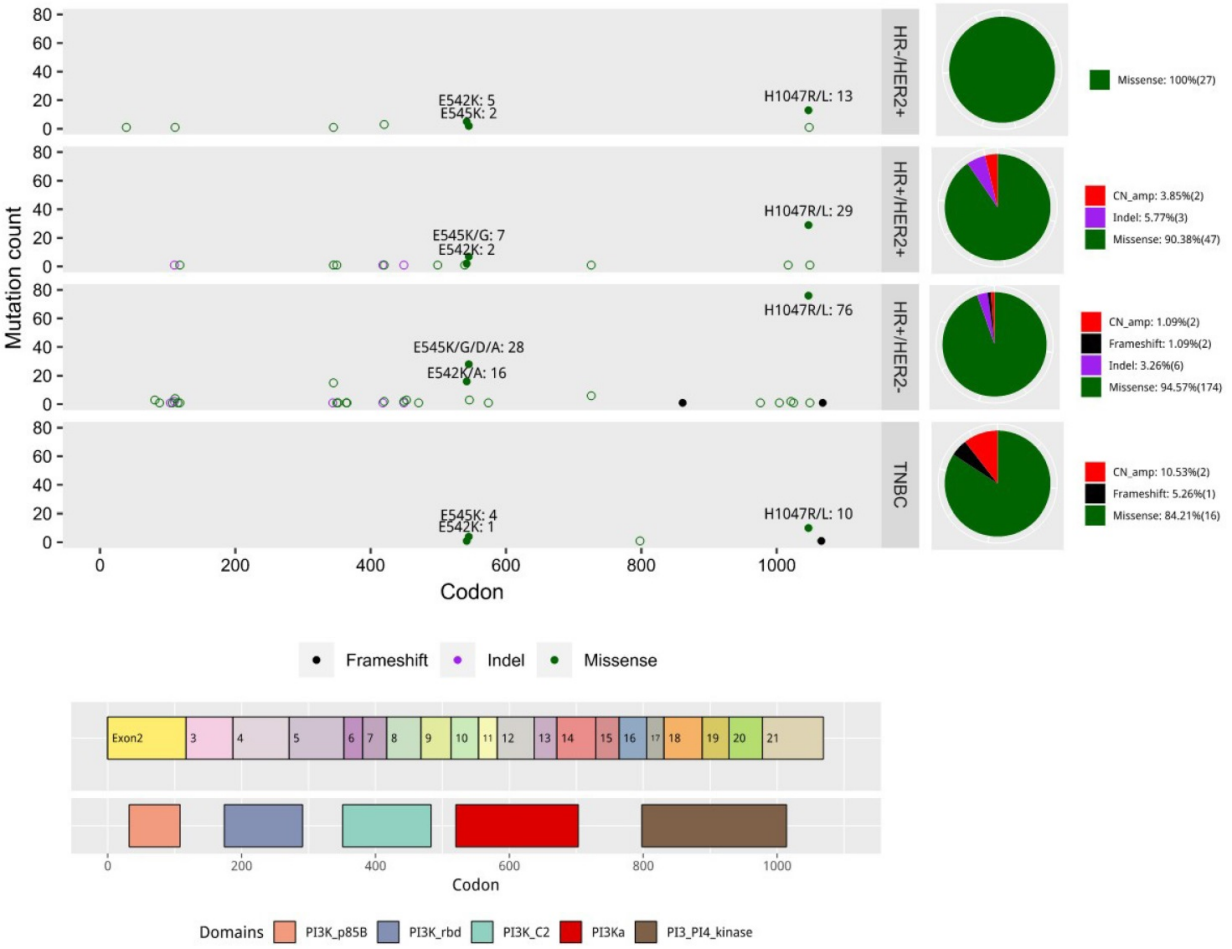
Distribution of *PIK3CA* mutation types in our cohort according to molecular subtypes. Colored boxes in the bottom indicate the five PIK3CA domains, including an adaptor binding domain (ABD), a Ras binding domain (RBD), a C2 domain, a helix domain, and a PI4K kinase catalytic domain. The distribution of mutations is according to specific mutation site. Each mutation type is indicated by color. The pie charts on the right summarize the distribution of mutation types for each molecular subtype.

A total of 265 patients had *PIK3CA* mutations, including 301 mutations, with some patients harboring more than 1 (multiple) mutations. Missense mutations accounted for 93.0% (280/301) of the *PIK3CA* mutations. The *PIK3CA* mutation frequency significantly differed among the four breast cancer molecular subtypes, with 48.3% (155/321) in HR^+^/HER2^-^, 45% (50/111) in HR^+^/HER2^+^, 42.2% (27/64) in HR^-^/HER2^+^, and 25% (17/68) in HR^-^/HER2^-^ patients (Table 1). The mutation sites were differentially distributed among the subtypes (Figure 2). A total of 58 *PIK3CA* mutations were identified in our cohort; 47 of these mutations were detected in HR^+^/HER2^-^ subtype, 18 were in HR^+^/HER2^+^ subtype, 9 were in HR^-^/HER2^+^ subtype, and 7 were in the HR^-^/HER2^-^ subtype. Four mutations, namely E545K, H1047R, E542K, and H1047L, were consistently detected in all four subtypes. Two mutations, C420R and N345K, were detected in three subtypes except in TNBC. Seven mutations, namely E545G, E418_L422delinsV, E726K, E110del, G1049R, G118D, and D350G, were only detected in HR^+^/HER2^-^ and HR^+^/HER2^+^ subtypes. There were 40 *PIK3CA* mutations only detected in any one subtype. Of them, 32 mutations were only detected in HR^+^/HER2^-^ subtype, 4 mutations (S499F, P449_E453delinsQ, D1017V, and P539R) were only detected in HR^+^/HER2^+^ subtype, 2 mutations (E39K and H1048L) were only detected in HR^-^/HER2^+^ subtype, and 2 mutations (A1066fs and E798N) were only detected in the HR^-^/HER2^-^ subtype.

*PIK3CA* H1047R in exon 20 affecting the catalytic subunit of *PIK3CA* was the most commonly mutated site (41.9%, 126/301). Mutations in exon 9 that affect the helix binding domain, E545K (13.0%, 39/301) and E542K (7.6%, 23/301), were also identified in our cohort (Figure 2). In addition to these common and typical “major-mutant” hotspots, some “minor-mutant” sites, including N345K (6.0%), H1047L (4.0%), E726K (2.3 %), and C420R (2.0%), were also detected in our cohort.

As shown in Table 1, *PIK3CA* mutations were more likely to be detected in patients over 40 years old (P = 0.002), those who are postmenopausal (P = 0.004), and had ER-positive (P = 0.002), and PR-positive (P = 0.012) types of breast cancer. Interestingly, the proportion of patients with *PIK3CA* mutations having Ki67 scores of >14% was significantly lower than that of *PIK3CA* wild-type (WT) patients. However, there were no significant differences between *PIK3CA*-mutant and WT patients in terms of HER2 status, tumor size, and lymph node metastasis (Table 1).

### Double PIK3CA-mutant tumors are frequent in breast cancer

As described above, 265 *PIK3CA*-mutant tumors were identified in our cohort. Among them, 34 (12.8%) cases harbored more than one *PIK3CA* mutations, and a vast majority (94.1%, 32/34) of such tumors had two *PIK3CA* mutations. In addition, the most common double *PIK3CA* mutation detected in our cohort consisted of a typical hotspot mutation (such as E542, E545, or H1047) and a second minor site. Tumors that harbored multiple *PIK3CA* mutations were HR-positive; of which, 5.9% (2/34) had HR^+^/HER2^+^ subtype.

### PTEN mutations

A total of 48 *PTEN* mutations were detected from 44 patients, with 4 patients detected with double *PTEN* mutations. Frame-shift mutations (50%, 24/48) and missense mutations (16.7%, 8/48) were the two most common *PTEN* mutation types. The mutation frequencies of *PTEN* were also significantly different across breast cancer molecular subtypes. *PTEN* mutations were predominantly detected among HR^+^/HER2^-^ subtype (77.3%, 34/44), followed by TNBC subtype (18.2%, 8/44). Only a very low percentage of *PTEN* mutated cases were HR^-^/HER2^+^ (2.27%, 1/44) and HR^+^/HER2^+^ (2.27%, 1/44) subtypes. No specific hotspot mutations were observed for *PTEN*, reflecting the diversity of mutation sites (Figure S2). Breast cancers with *PTEN* mutations were more likely to be HER2-negative. However, no significant differences were observed between *PTEN*-mutant and WT patients in terms of age, ER status, PR status, tumor size, lymph node metastasis, and Ki67 scores (Table 2).

### AKT1 mutations

In our cohort, a total of 35 patients were identified to harbor 36 *AKT1* mutations. The majority (94.3%, 33/35) of *AKT1* mutations were detected among patients with HR^+^/HER2^-^ breast tumor. *AKT1* mutations were predominantly missense mutations (91.7%, 33/36), and the remaining 8.3% (3/36) were copy number amplification. *AKT1* E17K was the hotspot mutation, accounting for 86.1% (31/36, Figure S3). Breast cancers with *AKT1* mutations were more likely to be ER-positive (P = 0.016), PR-positive (P = 0.002), and HER2-negative (P = 0.001). However, patients with *AKT1* mutations did not statistically differ from *AKT1* WT patients in terms of age, tumor size, lymph node metastasis, and Ki67 score (Table 3).

### Coexistence of PIK3CA, PTEN, and AKT1 mutations

As shown in Figure S4, 5.3% of patients (14 of 265) with *PIK3CA* mutation also had concurrent *PTEN* mutation; 1.1% of patients (3 of 265) with *PIK3CA* mutation also had concurrent *AKT1* mutation. No patients were detected with concurrent *PTEN* and *AKT1* mutations.

### Clinical significance of PI3K-AKT-mTOR pathway mutations

**Figure 3:**
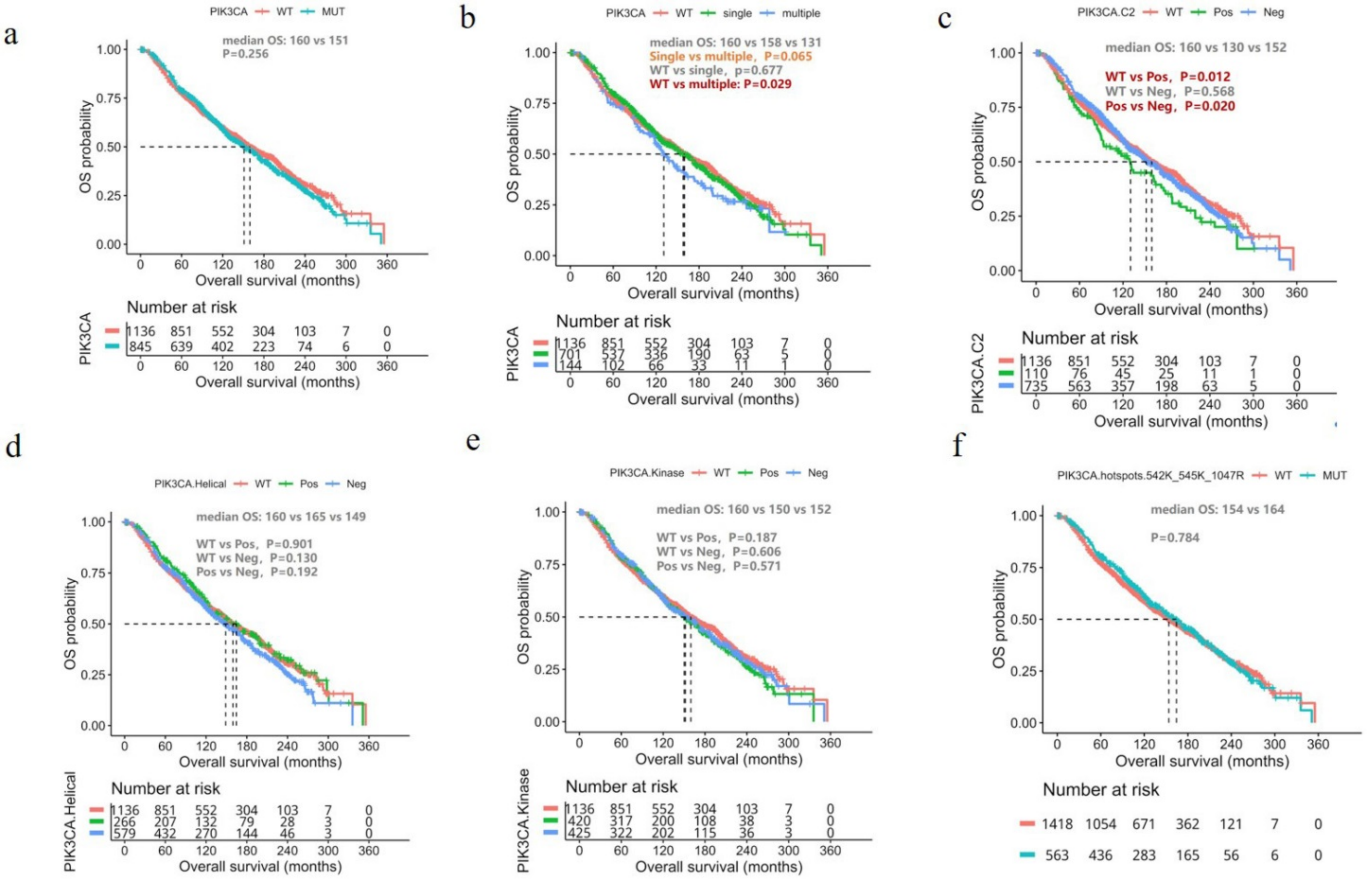
Prognostic impact of *PIK3CA* mutations. Kaplan Meier curves comparing the overall survival of the breast cancer cohort from the METABRIC dataset according to (**A**) The presence/absence of *PIK3CA* mutation (*PIK3CA*-mut vs. *PIK3CA*-WT); (**B**) The number of *PIK3CA* mutations (multiple, single, WT); (**C**) Mutations located in the PIK3CA-C2 domain vs. non-C2 domain; (**D**) Mutations located in the helical domain vs. non-helical domain; (**E**) Mutations in the kinase domain vs. non-kinase domain; (**F**) H1047R vs. non-H1047R mutations. WT, wild-type.

Since survival data for our cohort is unavailable, we analyzed the molecular and survival data of the breast cancer cohort from the METABRIC dataset 17. In general, OS was similar between patients with and without *PIK3CA* mutations (mOS, 152 vs. 159 months, P=0.256, Figure 3A). We then divided the patients with *PIK3CA* mutations into two groups according to the number of *PIK3CA* mutations they harbored: those with single and multiple *PIK3CA* mutations. Patients with multiple *PIK3CA* mutations had significantly shorter OS than those with *PIK3CA* WT (mOS, 131 vs. 159 months, P=0.029, Figure 3B) and a trend of shorter OS, but statistical significance was not reached, than those with single *PIK3CA* mutation (mOS, 131 vs. 162 months, P=0.065, Figure 3B). We also performed a subgroup analysis based on the location of the *PIK3CA* mutations. Interestingly, we found that patients with *PIK3CA* mutations located in the C2 domain had significantly shorter OS than patients with non-C2 domain mutations (mOS, 130 vs. 154 months, P=0.020, Figure 3C). However, there was no significant difference in prognosis between patients with non-C2 domain mutations and patients with WT *PIK3CA* (mOS, 152 vs. 160 months, P=0.568, Figure 3C). There was no difference observed in survival between patients with *PIK3CA* mutations located in either the helical domain (Figure 3D) or kinase domain (Figure 3E). Finally, patients with *PIK3CA* H1047R mutations also had significantly shorter OS than patients with non-H1047 mutations (mOS, 152 vs. 157 months, P=0.021, Figure 3F).

## Discussion

In this study, we performed a comprehensive analysis of the genetic aberrations associated with the PI3K-AKT-mTOR pathway in a large cohort of Chinese women with early-stage breast cancer (n = 589). Based on our results, *PIK3CA* was the most frequently mutated gene (45.0%), which is consistent with a similarly high frequency (43.6%) reported by Chen et al. 9, but numerically higher than the data from the Cancer Genome Atlas (TCGA) breast cancer dataset (36%) 20. Similar to previous reports 9, 20, the *PIK3CA* hotspot mutations observed in our cohort were also H1047R, E545K, and E542K mutations. Among them, the H1047R substitution in exon 20 accounted for 41.9% (126/301), the E545K substitution in exon 9 accounted for 13.0% (39/301), and the E542K substitution in exon 9 accounted for 7.6% (23/301) of all the *PIK3CA* mutations. Since H1047 is in the kinase domain and near the C-terminus of the activation loop, the substitution of histidine (H) to arginine (R) at this position may alter the conformation of the activation loop and result in the activation of the PI3K pathway 21, 22. H1047R mutation is sufficient to induce tumors in animal models and may be associated with a favorable response to PI3K inhibitors 21, 22. On the other hand, E545 is within the helical domain and interacts with the N-terminal SH2 domain of the p85 regulatory subunit 23. The change from glutamic acid (E) to lysine (K) causes charge reversal and disrupts its inhibitory interaction with p85 24. In addition to the three common and typical “major-mutant” hotspots (E542K, E545K, or H1047R), we also identified some “minor-mutant” sites, including N345K, H1047L, E726K, and C420R. Consistent with our findings, Martínez-Sáez et al. previously reported a *PIK3CA* mutation rate of 35.7% in their breast cancer cohort, with H1047R (35%), E545K (17%), E542K (11%), N345K (6%), and H1047L (4%) as the most common mutation sites 25. H1047L and N345K mutations are located in the kinase domain with relatively higher mutation frequency, and N345K and C420R mutations are located in the PIK3CA C2 domain, which are relatively rare.

In addition, we found that 12.8% of patients had two or more *PIK3CA* mutations. This data is also consistent with a previous report by Vasan et al 26. Analysis of 3,740 *PIK3CA*-mutated tumors identified about 12% of patients who harbored multiple *PIK3CA* mutations 26. The frequency of multiple *PIK3CA* mutations in our cohort is also largely consistent with those reported in the breast cancer cohort of the METABRIC dataset (13%) 27 and the TCGA dataset (11%) 20. In the METABRIC and TCGA datasets, a vast majority of breast tumors (88% to 96%) with multiple *PIK3CA* mutations also had double mutations. Through *in vivo* and *in vitro* experiments, Vasan et al. demonstrated that the presence of double *PIK3CA* mutations on the same allele resulted in increased PI3K activity, which led to enhanced downstream signal transduction, cell proliferation, and tumor growth 26. The molecular mechanism of *PIK3CA* double mutation includes increasing the destruction of the binding of p110α to the inhibitory subunit p85α, thereby reducing its catalytic inhibitory effect and enhancing the binding of p110α membrane lipids. As compared to single mutations, double *PIK3CA* mutations activate PI3K signaling pathway to a higher degree and promote the malignant progression of tumors 26. This can explain, to a certain extent, the poorer prognosis of patients with multiple *PIK3CA* mutations than those with only one *PIK3CA* mutation.

In our cohort, women with *PIK3CA* mutations were more likely to be older than 40 years of age (P = 0.002), postmenopausal (P = 0.004), ER-positive (P = 0.002), and PR-positive (P = 0.012). Among patients with *PIK3CA* mutations, the proportion of patients with Ki67 score of >14% was significantly lower than that of WT patients (Table 1). However, there were no significant differences between patients with or without *PIK3CA* mutations in terms of HER2 status, tumor size, and lymph node metastasis. Consistent with our findings, a pooled analysis of 10,319 women with early-stage breast cancer from 19 studies showed a *PIK3CA* mutation rate of 32% with the detection of *PIK3CA* mutations significantly associated with ER-positive status, increased age, lower tumor grade, and smaller body size (all P <0.001) 28. Tao et al. sequenced the plasma of 234 Chinese women with metastatic breast cancer and reported a *PIK3CA* mutation frequency of 31%, which were significantly higher in HR^+^/HER2^+^, HR^+^/HER2^-^, and HR-/HER2^+^ subtypes and significantly less common in TNBC (P<0.01) 29. Our results also found that *PIK3CA* mutations were the least in TNBC than the other subtypes.

In the PI3K-AKT-mTOR pathway, our study identified *PTEN* as the second most commonly mutated gene. *PTEN* mutations occurred in 7.5% (44/589) of patients, and the incidence rate of double-*PTEN* mutations was 9.09% (4/44). Millis et al. reported a *PTEN* mutation rate of 6% across 19,784 diverse solid tumors 1, while the *PTEN* mutation rate was about 10% in the TCGA cohort 20. However, the incidence of negative PTEN expression using immunohistochemistry of breast tumors was reported to be as high as 30% 1, which suggests that somatic mutations in *PTEN* are only a small proportion of breast tumors with PTEN loss-of-function. In our cohort, we observed a difference in the subtype distribution of *PTEN* and* PIK3CA* mutations. *PTEN* mutations occurred most frequently in HR^+^/HER2^-^ subtype, and second most in TNBC subtype. In addition, for* PTEN* mutations also lack typical hotspot mutations. Since *PTEN* is a tumor suppresser gene, any loss-of-function mutation would inactivate it in tumors. However, in the case of *PIK3CA,* only a few gain-of-function mutations would be selected during tumorigenesis. Another interesting observation in our cohort is that breast cancers with *PTEN* mutations were more likely to be HER2-negative, while no significant differences between patients with/without *PTEN* mutations in terms of age, ER status, PR status, tumor size, lymph node metastasis, and Ki67 score.

*AKT1* (5.9%) ranks as the third most commonly mutated gene in the PI3K-AKT-mTOR signaling pathway in our cohort. Almost all (94.3%, 33/35) of *AKT1* mutations were identified in HR^+^/HER2^-^ breast tumors, with only one typical hotspot mutation. *AKT1* E17K is a single amino acid mutation in the pleckstrin homology (PH) domain of the AKT1 protein, which has been reported to function as a carcinogenic driver mutation in breast cancer and other solid cancers 18, 19. In our cohort, breast cancers with *AKT1* mutations were significantly associated with positive ER- (P = 0.016) and PR (P = 0.002) but negative HER2- (P = 0.001) status. However, women with/without *AKT1* mutations were similar in terms of age, tumor size, lymph node metastasis, and Ki67 score.

The prognostic impact of *PIK3CA* mutation in breast cancer is still controversial. Several studies have shown that breast cancer patients with *PIK3CA* mutations have a better prognosis 30, 31, while Sobhani et al. reported a significantly worse prognosis for women with *PIK3CA*-mutant breast tumors 32. Gerratana et al. analyzed 88 cases of metastatic breast cancer by multivariate logistic regression and found that the detection of *PIK3CA* mutation in circulating tumor DNA was associated with lung metastasis (OR 3.74) 33. Our findings using the METABRIC dataset may partly explain this controversy. In our survival analysis, we found that patients with multiple *PIK3CA* mutations, but not those with single *PIK3CA* mutations, had significantly worse prognosis than patients with WT *PIK3CA*. In addition, we also found that patients with mutations in the PIK3CA-C2 domain had significantly worse prognosis than patients with non-C2 domain mutations (mOS, 130 vs. 154 months, P=0.020, Figure 3C). Furthermore, patients with *PIK3CA* H1047R mutation also had worse prognosis than patients without H1047 mutations (mOS, 152 vs. 157 months, P=0.021, Figure 3F). However, prognosis was comparable between patients with non-C2 domain mutations and WT *PIK3CA* (mOS, 154 vs. 159 months, P=0.568, Figure 3C). These results suggest the distinct prognostic impact of the number and position of *PIK3CA* mutations.

There are some limitations in our study. First, since we do not have the long-term follow-up data for our cohort, correlation analysis for long-term survival outcomes and genetic data were only performed using publicly available METABRIC dataset. Second, as our current study did not include data on protein expression or other functional studies, the actual impact of the individual gene mutation on protein products and the activation status of the PI3K-AKT-mTOR signaling pathway largely rely on speculation. Third, most of the patients in our cohort did not receive inhibitors targeting the PI3K-AKT-mTOR signaling; hence, the therapeutic implication of the analyzed gene mutations was not evaluated.

In conclusion, our study demonstrated that the genetic alteration of the PI3K-AKT-mTOR pathway gene is very common in breast cancer, with a mutation rate of 62.6%. The frequencies and types of mutations in the PI3K-AKT-mTOR pathway genes were distinct across the breast cancer molecular subtypes. In addition, survival analysis using the data from the METABRIC cohort demonstrated the prognostic impact of different mutation sites and the number of mutations in *PIK3CA*. Nevertheless, the therapeutic value of the PI3K-AKT-mTOR pathway using various specific inhibitors warrants further exploration and deeper investigation of the genetic mutational landscape of the pathway.

## Supplementary Material

Supplementary figure and tables.

## Figures and Tables

**Table 1:** Clinicopathological features of patients according to *PIK3CA* mutations.

Variables	Number of patients (n=589)	*PIK3CA* mutation		P-value
		Positive (n=265)	Negative (n=324)	
**Age (median 58,** **range 26-90)**				0.002
**≤40 years**	130	43(16.2%)	87(26.9%)	
**>40 years**	459	222(83.8%)	237(73.1%)	
**Menopausal status**				0.004
**Premenopause**	336	134(50.6%)	202(62.3%)	
**Postmenopause**	253	131(49.4%)	122(37.7%)	
**Tumor (T) stage**				0.409
**T1**	228	107(40.4%)	121(37.3%)	
**T2**	327	146(55.1%)	181(55.9%)	
**T3**	24	7(2.6%)	17(5.2%)	
**T4**	10	5(1.9%)	5(1.5%)	
**Lymph node metastasis**				0.316
**Negative (N0)**	253	120(45.3%)	133(41.0%)	
**Positive (N1-3)**	336	145(54.7%)	191(59.0%)	
**ER status**				0.000
**Negative**	155	51(19.2%)	104(32.1%)	
**Positive**	434	214(80.8%)	220(67.9%)	
**PR status**				0.012
**Negative**	183	68(25.7%)	115(35.5%)	
**Positive**	406	197(74.3%)	209(64.5%)	
**HER-2/neu status**				0.149
**Negative**	389	172(64.9%)	217(67.0%)	
**Positive**	175	77(29.1%)	98(30.2%)	
**Equivocal**	25	16(6.0%)	9(2.8%)	
**Subtypes**				0.003
**HR^+^/HER2^-^**	321	155(56.5%)	166(51.2%)	
**HR^+^/HER2^+^**	111	50(18.9%)	61(18.8%)	
**HR^-^/HER2^+^**	64	27(10.2%)	37(11.4%)	
**HR^-^/HER2^-^**	68	17(6.4%)	51(15.7%)	
**Unknown**	25	16(6.0%)	9(2.8%)	
**Ki67 score**				0.001
**≤ 14%**	137	80(30.2%)	57 (17.6%)	
**>14%**	448	184(69.4%)	264(81.5%)	
**Unknown**	4	1(0.4%)	3(0.9%)	

**Table 2:** Clinicopathological features of patients according to *PTEN* mutations.

Variables	Number of patients (n=589)	*PTEN* mutation		P-value
		Positive (n=44)	Negative (n=545)	
**Age (median 58,** **range 26-90)**				0.705
**≤40 years**	130	8(18.2%)	122(22.4%)	
**>40 years**	459	36(81.8%)	423(77.6%)	
**Menopausal status**				0.429
**Premenopause**	336	28(63.6%)	308(56.5%)	
**Postmenopause**	253	16(36.4%)	237(43.5%)	
**Tumor (T) stage**				0.622
**T1**	228	16(36.4%)	212(38.9%)	
**T2**	327	25(56.8%)	302(55.4%)	
**T3**	24	3(6.8%)	21(3.9%)	
**T4**	10	0(0.0%)	10(1.8%)	
**Lymph node metastasis**				0.268
**Negative (N0)**	253	15(34.1%)	238(43.7%)	
**Positive (N1-3)**	336	29(65.9%)	307(56.3%)	
**ER status**				0.72
**Negative**	155	10(22.7%)	145(26.6%)	
**Positive**	434	34(77.3%)	400(73.4%)	
**PR status**				0.402
**Negative**	183	11(25.0%)	172(31.6%)	
**Positive**	406	33(75.0%)	373(68.4%)	
**HER-2/neu status**				
**Negative**	389	42(95.5%)	347(63.7%)	0.000
**Positive**	175	2(4.5%)	173(31.7%)	
**Equivocal**	25	0(0.0%)	25(4.6%)	
**Subtypes**				0.002
**HR^+^/HER2^-^**	321	34(77.3%)	287(52.7%)	
**HR^+^/HER2^+^**	111	1(2.3%)	110(20.2%)	
**HR^-^/HER2^+^**	64	1(2.3%)	63(11.6%)	
**HR^-^/HER2^-^**	68	8(18.2%)	60(11.0%)	
**Unknown**	25	0(0.0%)	25(4.6%)	
**Ki67 score**				0.385
**≤ 14%**	137	11(25.0%)	126(23.1%)	
**>14%**	448	32(72.7%)	416(76.3%)	
**Unknown**	4	1(2.3%)	3(0.6%)	

**Table 3:** Clinicopathological features of patients according to *AKT1* mutations.

Variables	Number of patients (n=589)	*AKT1* mutation		P-value
		Positive (n=35)	Negative (n=554)	
**Age (median 58, range 26-90)**				0.205
**≤40 years**	130	11(31.4%)	435(78.5%)	
**>40 years**	459	24(68.6%)	119(21.5%)	
**Menopausal status**				0.164
**Premenopause**	336	24(68.6%)	312(56.3%)	
**Postmenopause**	253	11(31.4%)	242(43.7%)	
**Tumor (T) stage**				0.157
**T1**	228	19(54.3%)	209(37.7%)	
**T2**	327	15(42.9%)	312(56.3%)	
**T3**	24	0(0.0%)	24(4.3%)	
**T4**	10	1(2.9%)	9(1.6%)	
**Lymph node metastasis**				0.168
**Negative (N0)**	253	11(31.4%)	242(43.7%)	
**Positive (N1-3)**	336	24(68.6%)	312(56.3%)	
**ER status**				0.016
**Negative**	155	3(8.6%)	152(27.4%)	
**Positive**	434	32(91.4%)	402(72.6%)	
**PR status**				0.002
**Negative**	183	3(8.6%)	180(32.5%)	
**Positive**	406	32(91.4%)	374(67.5%)	
**HER-2/neu status**				0.001
**Negative**	389	33(94.3%)	356(64.3%)	
**Positive**	175	2(5.7%)	173(31.2%)	
**Equivocal**	25	0(0.0%)	25(4.5%)	
**Subtypes**				0.002
**HR^+^/HER2^-^**	321	33(94.3%)	288(52.0%)	
**HR^+^/HER2^+^**	111	2(5.7%)	111(20.0%)	
**HR^-^/HER2^+^**	64	0(0.0%)	62(11.2%)	
**HR^-^/HER2^-^**	68	0(0.0%)	68(12.3%)	
**Unknown**	25	0(0.0%)	25(4.5%)	
**Ki67 score**				0.385
**≤ 14%**	137	15(42.9)	122(22.0)	
**>14**	448	20(57.1)	428(77.3)	
**Unknown**	4	0(0.0)	4(0.7)	

## Abbreviations

AKT: protein kinase B
BC: breast cancer
ER: Estrogen receptor
FFPE: formalin-fixed paraffin-embedded
HER2: human epidermal growth factor receptor 2
HR: Hormone receptors
M: metastasis
mOS: median overall survival
mTOR: mammalian target of rapamycin
N: lymph node involvement/metastasis
NGS: next-generation sequencing
OS: overall survival
PFS: progression-free survival
PI3K: phosphoinositide 3-kinase
PIP_3_: phosphatidylinositol 3,4,5-triphosphate
PR: Progesterone receptor
PTEN: phosphatase and tensin homolog
RTK: receptor tyrosine kinase
T: tumor
TNBC: triple-negative breast cancer
WT: wild-type
