# Supplementary material for: Mutational Landscape of PI3K-AKT-mTOR Pathway in Breast Cancer: Implications for Targeted Therapeutics
Source: J Cancer. 2021 May 27;12(14):4408–17. doi: 10.7150/jca.52993 (PMC8176410; doi:10.7150/jca.52993)
Supplement: Supplementary file 1 — Supplementary figure and tables. [file jcav12p4408s1.pdf]

Supporting Information

Table S1. The clinicopathological characteristics of 589 women with breast cancer in our cohort

**Characteristics**		**No.(%)**
**Age**	>40 years	459(77.9%)
	≤40 years	130(22.1%)
**Menopausal status**	Pre-menopausal	336(57.0%)
	Post-menopausal	253(43.0%)
**Pathological stage**	I	137(23.3%)
	II	329(55.9%)
	III	123(20.9%)
**Pathological tumor (T) stage**	T1	228(38.7%)
	T2	327(55.5%)
	T3	24(4.1%)
	T4	10(1.7%)
**Pathological lymph node (N) stage**	N0	253(43.0%)
	N1	220(37.4%)
	N2	82(13.9%)
	N3	34(5.8%)
**Pathological metastasis (M) stage**	M0	589(100.0%)
**Histological grade**	I	22(3.7%)
	II	274(46.5%)
	III	282(47.9%)
	Unknown	11(1.9%)
**HR/HER2 status**	HR-/HER2-	68(11.5%)
	HR-/HER2+	64(10.9%)

	HR+/HER2-	321(54.5%)
	HR+/HER2+	111(18.8%)
	Unknown	25(4.2%)
**Histological type**	DCIS	8(1.4%)
	IDC	514(87.3%)
	ILC	14(2.4%)
	Other	53(9.0%)
**Ki67 status**	> 14%	448(76.1%)
	≤ 14%	137(23.3%)
	Unknown	4(0.7%)

Abbreviation: DCIS: ductal carcinoma in situ; HER2: human epidermal growth factor receptor 2; HR: hormone receptor; IDC: invasive ductal carcinoma; ILC: invasive lobular carcinoma

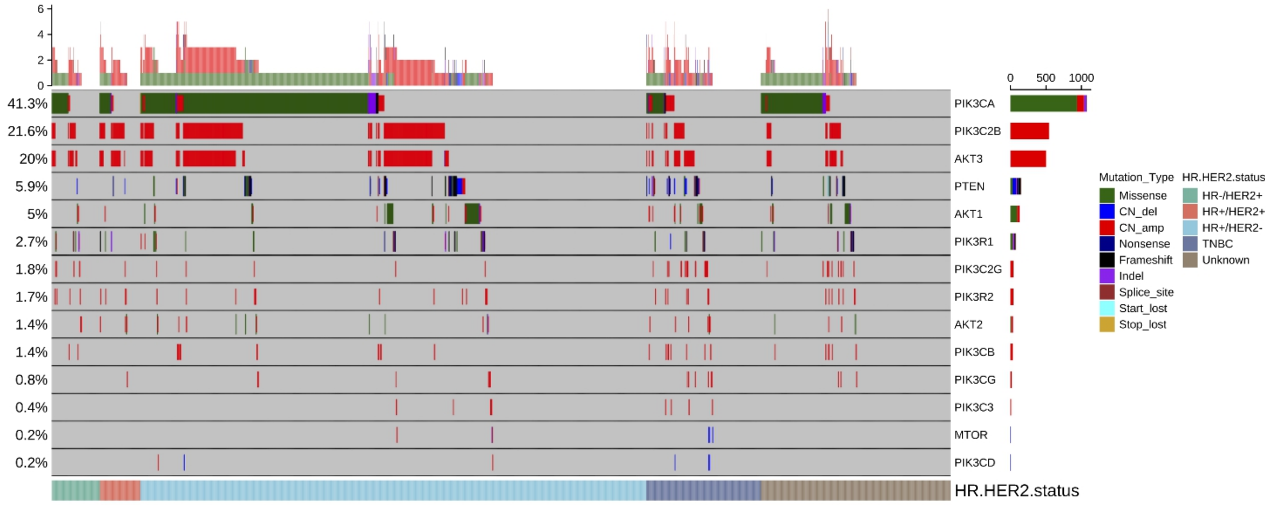

Figure S1. Mutation profile of genes in the PI3K-AKT-mTOR pathway of 2,509 breast cancer cohort of the METABRIC dataset. Tumor samples were grouped by molecular subtype: HR+/HER2- (n =1413), HR+/HER2+ (n =113), HER2-rich (n =134), and TNBC (n =320) as indicated by the annotation at the bottom. The mutation frequency for each gene was shown on the left. Colors indicated the mutation types.

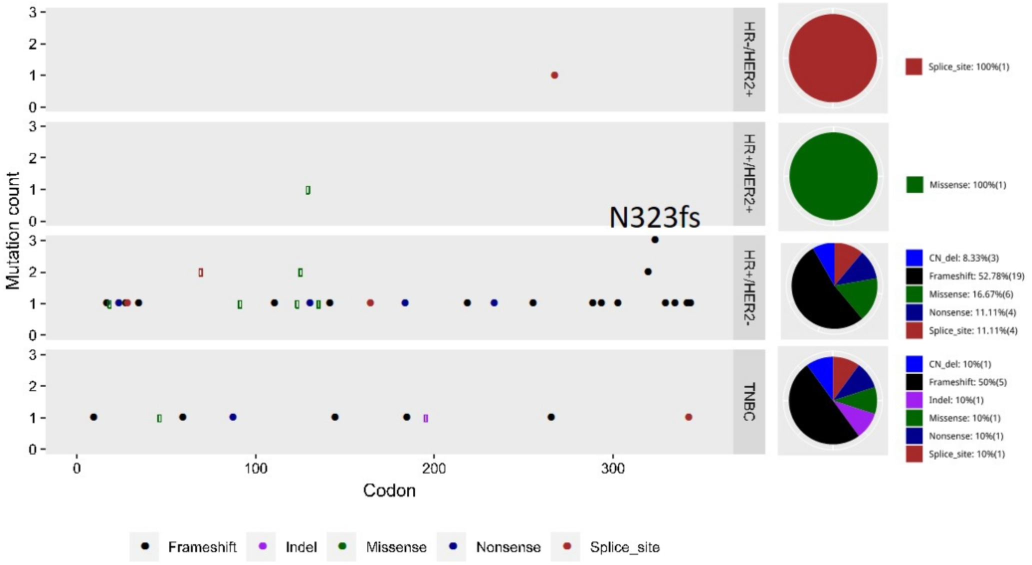

Figure S2. Distribution of *PTEN* mutation types in our cohort according to molecular subtypes. The distribution of mutations is according to specific mutation site. Each mutation type is indicated by color. The pie charts on the right summarize the distribution of mutation types for each molecular subtype.

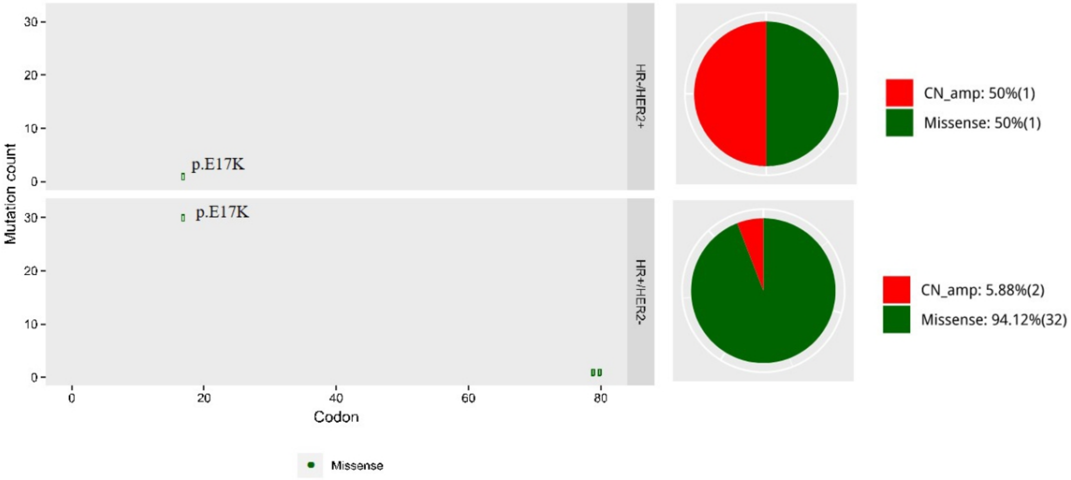

Figure S3. Distribution of *AKT1* mutation types in our cohort according to molecular subtypes. The distribution of mutations is according to specific mutation site. Each mutation type is indicated by color. The pie charts on the right summarize the distribution of mutation types for each molecular subtype.

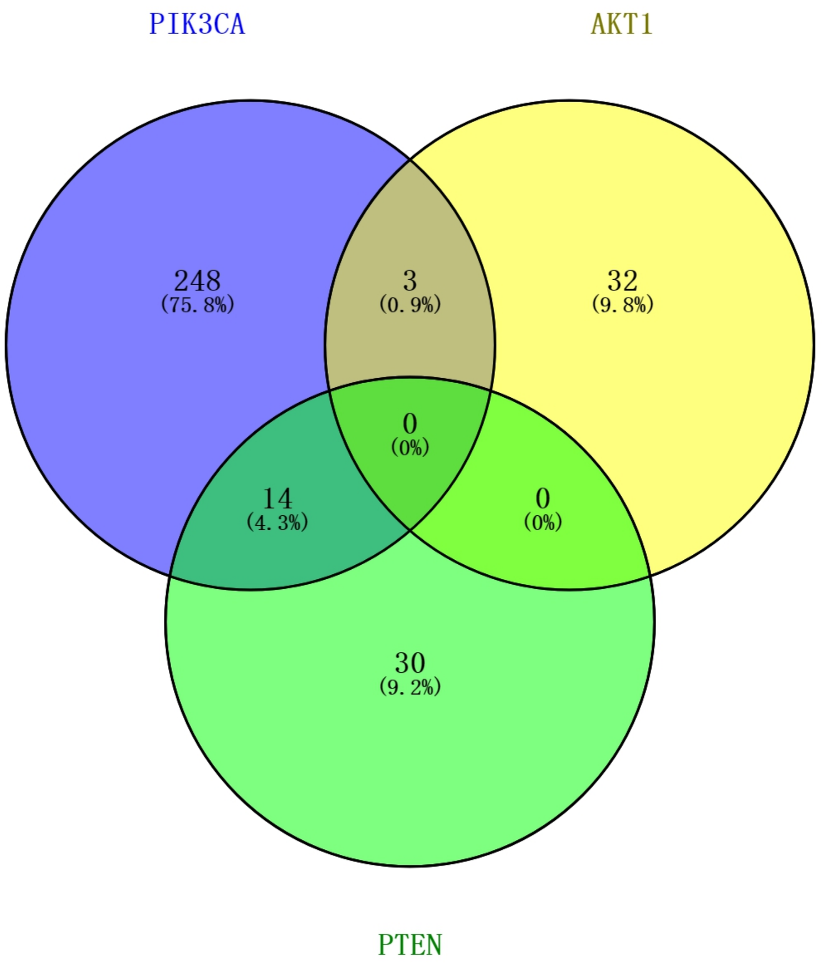

Figure S4. The Venn diagram shows the co-occurrence of *PIK3CA*, *PTEN*, and *AKT1* mutations. Of the 265 breast cancer patients with *PIK3CA* mutations, 14 had *PTEN* mutations and 3 had *AKT1* mutations, and the remaining 248 patients had neither *PTEN* mutations nor *AKT1* mutations.
